# AI-Assisted Diagnostics in Dentistry: An Eye-Tracking Study on User Behavior

**DOI:** 10.4317/jced.61491

**Published:** 2024-05-01

**Authors:** Laura Winterhalter, Florian Kofler, Dragan-Alexander Ströbele, Ahmed Othman, Constantin von See

**Affiliations:** 1 Undergraduate dental student, Danube Private University, Krems, Austria; 2 Helmholtz AI, Helmholtz Munich, Neuherberg, Germany; 3 Department of Computer Science, TUM School of Computation, Information and Technology, Technical University of Munich, Munich, Germany; 4 TranslaTUM - Central Institute for Translational Cancer Research, Technical University of Munich, Munich, Germany; 5 Department of Diagnostic and Interventional Neuroradiology, School of Medicine, Klinikum rechts der Isar, Technical University of Munich, Munich, Germany; 6 Assistant Professor in the digital technologies in dentistry and CAD/CAM department- Danube Private University- Austria; 7 Assistant professor and senior orthodontist in the digital technologies in dentistry and CAD/CAM department- Danube Private University- Austria; 8 Director of the digital technologies in dentistry and CAD/CAM department- Danube Private University- Austria

## Abstract

**Background:**

Artificial Intelligence (AI) has increasingly been integrated into dental practices, notably in radiographic imaging like Orthopantomograms (OPGs), transforming diagnostic protocols. Eye tracking technology offers a method to understand how dentists’ visual attention may differ between conventional and AI-assisted diagnostics, but its integration into daily clinical practice is challenged by the cost and complexity of traditional systems.

**Material and Methods:**

Thirty experienced practitioners and dental students participated to evaluate the effectiveness of two low-budget eye-tracking systems, including the Peye Tracker (Eye Tracking Systems LTD, Southsea, UK) and Webgazer.js (Brown University, Providence, Rhode Island) in a clinical setting to assess their utility in capturing dentists’ visual engagement with OPGs. The hardware and software setup, environmental conditions, and the process for eye-tracking data collection and analysis are illustrated.

**Results:**

The study found significant differences in eye-tracking accuracy between the two systems, with Webgazer.js showing higher accuracy compared to Peye Tracker (*p*<0.001). Additionally, the influence of visual aids (glasses vs. contact lenses) on the performance of eye-tracking systems revealed significant differences for both Peye Tracker (*p*<0.05) and Webgazer.js (*p*<0.05).

**Conclusions:**

Low-budget eye-tracking devices present challenges in achieving the desired accuracy for analyzing dentists’ visual attention in clinical practice, highlighting the need for continued innovation and improvement in this technology.

** Key words:**Artificial intelligence, Eye-tracking device, low-budget, dentistry.

## Introduction

Artificial Intelligence (AI) has progressively found its place within the realm of dentistry, offering revolutionary pathways for diagnosis, treatment planning, and patient care (1,2). As some of these technical opportunities are still under scientific investigation others have found their way in the daily routine of clinical practitioners.

Among its most noTable applications is in dental radiographic imaging, including lateral cephalometric X-rays, Bitewing, and Orthopantomogram (OPG) analyses (3-5). Using AI technologies implies a mathematical model to a given task. This procedure is completely disrupting the examination protocol for medical image analysis. Dentists traditionally approach the examination of every anatomical structure in an OPG with thorough attention, following a systematic protocol to ensure no detail is overlooked (6).

As AI technology becomes increasingly integrated into patient care, its impact on clinical decision-making processes remains still unexplored. Specifically, the question of whether dentists employ different analytical approaches to AI-annotated OPGs as opposed to non-annotated ones is yet to be examined.

To explore the potential difference in how dentists analyze AI-annotated versus non-annotated OPGs, validating a method to capture their visual attention patterns during analysis is essential. Eye tracking, which measures both the location and duration of gaze within a visual field, emerges as a crucial tool for this purpose, providing invaluable insights into visual attention in various disciplines (7,8).

In the context of dentistry, this technique could provide insights into the cognitive processes involved in diagnostic tasks, offering a unique perspective on how dental professionals interact with radiographic images in an everyday clinical scenario (9). However, integrating eye tracking into a clinical practice presents unique challenges, not least because of the high costs associated with scientific-grade eye-tracking equipment and the deviation from a classical scientific setting (10).

This pilot study addresses these obstacles by evaluating whether low-budget, commercially available eye-tracking devices can provide accurate and reliable data within a clinical practice to further evaluate the potential of AI annotation on human decision-making and dental diagnosis.

## Material and Methods

This study assesses the effectiveness and precision of two low-budget, commercially available eye-tracking systems in documenting dentists’ visual focus as they examine OPGs within the clinical practice.

Each system is evaluated based on its hardware and software specifications, implementation according to manufacturer instructions, eye-tracking equipment capabilities, and environmental setup (Table 1).

For this investigation two completely different eye-tracking systems have been investigated.

The first system is the eye-tracking device Peye Tracker (Eye Tracking Systems LTD, Southsea, UK). The device is compatible with the minicomputer Raspberry Pi and can be mounted on a display screen or other flat surfaces for flexible usage.

The setup of the Peye Tracker eye-tracking device, guided by manufacturer specifications, is optimized for accuracy in a clinical environment. It consists of a Windows 10 Professional Edition (Microsoft Corp., Redmond, USA) computer and a 24-inch Asus VE 248 (ASUSTeK Computer Inc, Taipei, Taiwan) screen with a 1920x1080 resolution, a 60 Hz refresh rate and RGB color format and a brightness of up to 250 cd/m².

Further, a Raspberry Pi Version 4B (Raspberry Pi Foundation, Cambridge, UK), equipped with the Peye Tracker capable of tracking at 50 fps, was utilized. Positioned 60 cm away from participants, it supports movements within an 18.5 cm by 30 cm area without compromising accuracy. This system, linked to a Windows computer via Peye Tracker Client software version 5.3 (Eye Tracking Systems LTD, Southsea, UK), wirelessly captures and records gaze data to a comma-separated values (CSV) file for easy processing.

The second platform in this study is Webgazer.js, an open-source, browser-based eye-tracking library developed by the WebGazer Team of Brown University (Providence, Rhode Island) that utilizes a laptop’s built-in or external camera to monitor gaze patterns, eliminating the need for specialized hardware.

It is implemented on a 2023 MacBook Air (Apple Inc., Cupertino, USA) with a 13-inch Retina display, offering a resolution of 2560x1600 pixels and luminance of 400 cd/m², on Mac OS Sonoma 14.2. in combination with an IPEVO V4K external camera, supporting 4k resolution and a frame rate of 30 fps, and operating through Mozilla Firefox Version 120.0 (Mozilla Foundation, Mountain View, CA).

The setup strictly follows developer guidelines and calibration to ensure a reliable eye-tracking environment, with preliminary assessments identifying the optimal setup to maintain the study’s methodological integrity, similar to the approach with the Peye Tracker.

The study is conducted in a setting designed to mimic the clinical practice in contrast to the usual scientific conditions for eye-tracking analysis, including measurements of illuminance ranging from 100 to 200 lux, no fixed head position, and relaxed, seated position in front of the eye-tracking systems while ensuring the reliability and accuracy of the data collected.

From a collection of pseudonymized patient radiographic images from the outpatient clinic (Danube Private University) ten OPG images depicting various dental conditions and hard tissue irregularities were chosen to be analyzed by the participants of this study, adhering to privacy and data protection guidelines.

Using the certified software “dentalXrai Pro” (dentalXrai GmbH, Berlin, Germany), the previously selected ten OPG images were annotated based on hard tissue specifics like crowns, carious lesions, implants, fillings, and root canal procedures. The AI annotations focused exclusively on hard tissue features, while soft tissues and any voids within the skull were not annotated and thus not regarded.

Experienced practitioners and dental students from the first clinical semester upwards at the Dental Clinic Krems of Danube Private University were voluntarily recruited to diagnose OPG images, leveraging their diverse levels of training and expertise.

The study divided participants into three groups (N=10) by visual aids: no aids (na), glasses (ag), and contact lenses (ac), highlighting voluntary participation, data anonymity, and minimal risks while ensuring privacy (Table 2).

On the Peye Tracker system, participants are seated directly in front of the monitor, positioning the eye tracker at the base of the screen as per the manufacturer’s guidelines, with eye alignment confirmed via a secondary display. Calibration requires participants to focus on and click nine points on the screen until they stop flashing, with encouragement to blink between points to minimize discomfort but to refrain from blinking during fixation. Following calibration, participants are shown a series of OPG images, initially without AI annotations, followed by versions with AI-enhanced details. The system records their gaze patterns for each image, using this data to generate heatmaps to visually represent the focus distribution across different image regions.

The Webgazer.js system setup requires positioning participants to ensure the webcam aligns with their eye level. Similar to the Peye Tracker system, the calibration process involves focusing on and clicking nine points, which shift from red to yellow upon successful calibration. The participants are advised to blink between fixations and blink multiple times before the final calibration step, which involves fixating on a central point for 10 seconds without blinking to enhance accuracy.

Calibration for both systems is performed per the manufacturer’s guidelines. In the clinical environment, participants maintain an upright yet relaxed posture while minimizing head movements to ensure accurate data capture. ComforTable seating is prioritized, although it comes at the cost of data consistency with the eye-tracking device.

Participants are allowed natural head movement, advised to sit comfortably with their legs forming a 90-degree angle to the floor, simulating the typical posture of a dentist reviewing radiographic images. This setup is intended to capture authentic diagnostic behaviors in a realistic clinical scenario without imposing unnatural postural restrictions.

After calibration, participants are shown a series of OPG images, first without and then with AI annotations. The system captures gaze patterns for each image, using this data to generate heatmaps that visually depict how visual attention was distributed across the images.

All CSV logs from the Peye Tracker are cleaned and mapped as x and y coordinates on a 300 DPI, 150%-scaled OPG image. Gaze data is transformed into heatmaps using Gaussian KDE in Python, showing visual focus areas.

Gaze data from the Webgazer.js system is stored on a SQL (Structured Query Language) database and rendered using an HTML document to display attention patterns.

The generated heatmaps do not include any axes or titles to maintain focus on the visualization of gaze data distribution.

-Statistical analysis 

Analysis of each eye-tracking system accuracy is conducted as part of a clinical practice pilot study and therefore precludes advance sample size calculation due to its preliminary nature.

Statistical comparisons between the two eye-tracking systems are performed using SigmaPlot 13.0 (Systat Software Inc., Chicago, USA). Furthermore, a separate analysis regarding visual aid usage (none, glasses, contact lenses) is conducted for both systems. The significance level is defined *p*<0.05.

## Results

The total of 30 candidates successfully participated without issues, and all collected data were deemed accurate and suitable for statistical analysis. The gender distribution is 70% male and 30% female, three groups are evaluated based on the necessity of visual aids - glasses (ag), contact lenses (ac) and no visual aids (na), consisting of ten individuals (N=10) each (Table 2).

While the Webgazer.js demonstrates significantly higher accuracy in eye tracking compared to the Peye Tracker, with mean accuracies of 75.1% ± 12.0% for Webgazer.js and 34.9% ± 27.0% for Peye Tracker.

The Mann-Whitney Rank Sum Test reveals a statistically significant difference between the groups (*p* < 0.001). While the Webgazer.js demonstrates significantly higher accuracy in eye tracking compared to the Peye Tracker, with mean accuracies of 75.1% ± 12.0% for Webgazer.js and 34.9% ± 27.0% for Peye Tracker.

**Figure 1:**
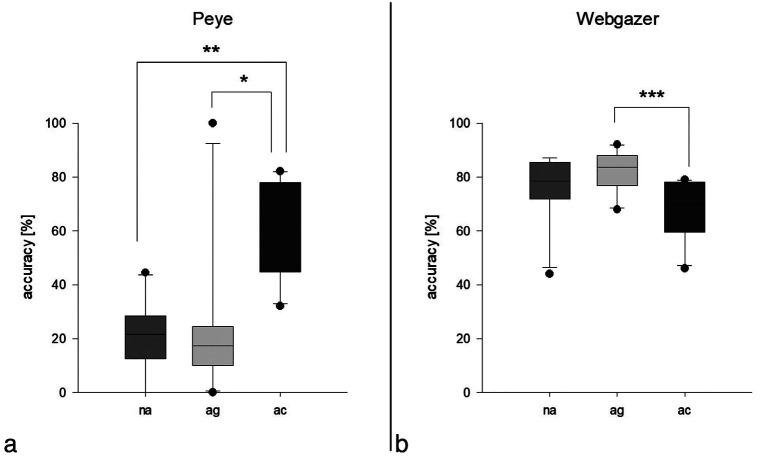
Accuracies based on groups; no visual aids (na), visual aids glasses (ag), visual aids contact lenses (ac), in Peye Tracker significantly higher accuracy for ac compared to ag (*, p=0.002) and to na (**, p=0.003), in Webgazer.js significantly higher accuracy for ag compared to ac (***, p=0.010).

The results (Fig. 1) demonstrated statistically significant differences in calibration accuracy for both the Peye tracker and Webgazer.js systems, indicating that visual aids substantially influence eye-tracking performance.

Normality testing using the Shapiro-Wilk test yields a *p-value* greater than 0.05, indicating a failure to reject the null hypothesis of normality. Consequently, a Mann-Whitney Rank Sum Test is conducted, revealing a statistically significant difference between the groups (*p* < 0.001), with Webgazer.js demonstrating significantly higher accuracy (Fig. 1).

The analysis regarding visual aids shows no normality for the Peye Tracker (*p*>0.05) or Webgazer.js (*p*>0.05). There are no statistically significant differences in the Peye Tracker (*p*<0.001) or Webgazer.js (*p*=0.013) subgroups.

**Figure 2:**
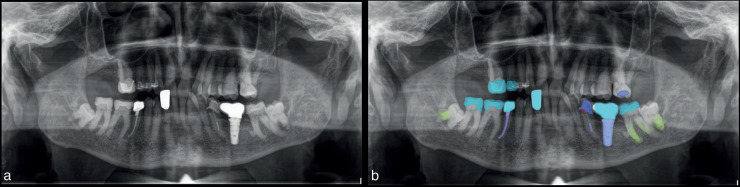
Participants view and analyze the original OPG (a) first and OPG with AI annotations (b) last while their eye movements are recorded with the Peye Tracker and Webgazer.js systems.

In case of the Peye Tracker, the group wearing contact lenses exhibit significantly higher accuracy compared to both the group wearing glasses (*p* = 0.002) and the group without visual aids (*p* = 0.003). No significant differences are found between the group without visual aids and the group wearing glasses (*p* = 0.991) (Fig. 2 left).

For Webgazer.js, the group wearing glasses display significantly higher accuracy compared to the group wearing contact lenses (*p* = 0.010). No other significant differences are observed among the groups (*p* > 0.05) (Fig. 2 right).

Participants initially examined the original OPG (Fig. 2a) and subsequently analyzed the AI-annotated OPG (Fig. 2b), with their eye movements recorded using both the Peye Tracker and Webgazer.js systems.

**Figure 3:**
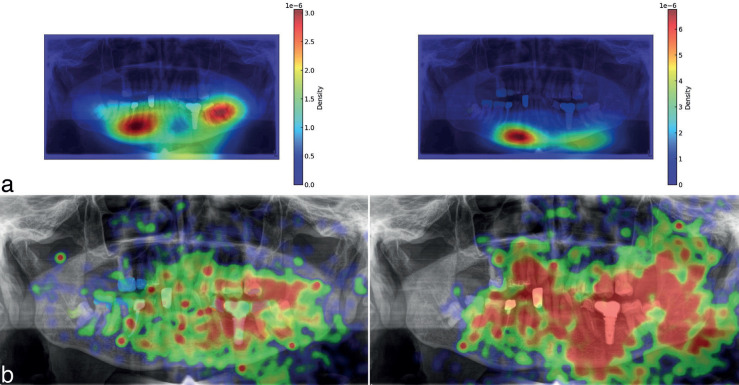
a) Heatmaps based on Peye Tracker gaze data illustrate the distribution of visual attention, indicating areas of diagnostic interest overlaid on OPG images (Original OPG (left) vs. AI-annotated (right)) at 60% transparency show gaze density using a color gradient, with warmer colors indicating higher fixation. The color bar indicates density values. b) Heatmaps based on Webgazer.js data display focal points of visual attention, highlighting regions of interest on the unannotated OPG (left) compared to the AI-annotated OPG (right), demonstrating variations in gaze behavior. In the heatmaps, pathologies such as the proximity of tooth number 48’s roots to the inferior alveolar nerve do not show a region of interest in either sets of OPG images.

The heatmaps based on the Peye Tracker gaze data illustrate the distribution of visual attention, indicating areas of diagnostic interest on the original OPG (Fig. 3a) and OPG with AI annotations (Fig. 3b), revealing differences in gaze patterns.

**Figure 4:**
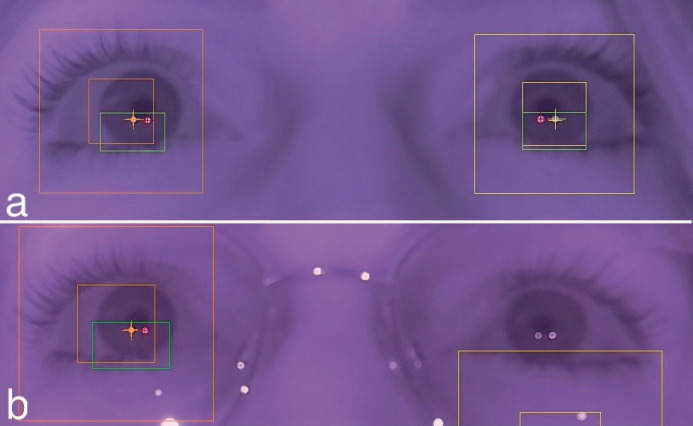
a) Real-time eye detection of a participant without visual aids (na) by the Peye Tracker, illustrating iris and pupil focus within yellow bounding boxes. Pupil movements are denoted by a green box, with pupil diameter indicated by yellow and red pointers. b) Real-time eye detection of a participant with glasses (ag) showing misidentification, where the left eye is not correctly detected due to the reflection from the metal frame of glasses, marked by a yellow box in the expected eye region.

The Peye Tracker’s real-time eye detection of a participant not using visual aids (na) captured the iris and pupil within yellow bounding boxes, highlighting pupil movements with a green box. Pupil diameter was further detailed using yellow and red pointers (Fig. 4a).

In the real-time eye detection trials, participants wearing glasses (ag) experienced misidentification issues, notably with the left eye not being accurately detected due to reflections from the glasses’ metal frame, as indicated by a yellow box in the anticipated eye region (Fig. 4b).

## Discussion

The integration of Artificial Intelligence (AI) in dentistry, particularly in radiographic imaging such as lateral cephalometric X-rays, Bitewing, and OPG analyses, represents a transformative advance in dental diagnostics and patient care (2).

This potential is further supported by studies such as those by Carrillo-Perez *et al*. (2022) and Shan, Tay, & Gu (2021), which explore the applications and performance of AI in various aspects of dentistry, including prosthodontics and clinical practice (11,12). The utilization of AI, as demonstrated by DentalXrai for AI annotation in OPG and Bitewing images, suggests a shift in how dental professionals approach radiographic analyses, potentially altering their diagnostic workflows (5).

Furthermore, the article by Schwendicke *et al*. (2021) adds to the discussion by highlighting the potential of AI to enhance diagnostic accuracy, patient outcomes, and efficiency in dentistry (2). The study emphasizes the importance of AI in identifying pathologies in radiographs with a level of precision that augments traditional diagnostic methods. It also points to the necessity of integrating AI tools seamlessly into dental workflows to harness their full potential while mitigating any possible resistance from dental professionals due to technological adjustments.

A critical evaluation into whether AI annotations influence dentists’ analysis of OPGs compared to traditional methods is yet to be thoroughly investigated. This deficiency points to the necessity for methods that effectively capture dentists’ visual engagement during the examination of OPGs.

Eye tracking technology, by measuring gaze patterns, not only sheds light on where dentists focus their attention but also serves as a valuable tool for delving into the cognitive processes that guide dental diagnostic practices, as Botelho *et al*. (2020) have noted (7).

The application of this technology across medical and dental education and diagnostics has been extensively researched, exemplified by Krupinski *et al*. (2010), who illustrated eye-tracking’s effectiveness in enhancing diagnostic precision by studying radiologists’ gaze behaviors during image assessments (13).

Moreover, the integration of eye tracking in dental education and diagnostics aligns with emerging trends in AI within dentistry, as discussed by Agrawal and Nikhade (2022) and Ahmed *et al*. (2021) (14,15).

These technologies offer a pathway to not only identifying areas of visual focus and potential oversight in dental imagery analysis but also to enhancing educational methodologies and diagnostic precision (16).

However, the impact of AI annotations on dentists’ diagnostic decisions remains unexplored.

Assessing these cost-effective eye-tracking solutions tackles key obstacles, including the substantial cost difference compared to research-grade eye-tracking systems and the difficulty of incorporating this technology into dental operations in various settings in clinical everyday life.

This study evaluated the Peye Tracker and Webgazer.js eye-tracking systems in dental diagnostics, highlighting their cost-effectiveness and potential to improve diagnostic accuracy as well as applicability within a clinical practice. However, challenges include specialized technical expertise, time-intensive setup of the eye trackers, and limited feasibility in clinical settings due to the little accuracy of the results achieved.

By examining the performance of the Peye Tracker and Webgazer.js systems, this research aims to contribute valuable insights into the feasibility of employing more accessible eye-tracking solutions in dental diagnostics.

The assessment of the Peye Tracker and Webgazer.js systems in this study was comprehensive, covering hardware and software specifications, implementation fidelity to manufacturer instructions, equipment capabilities, and environmental setup considerations. The methodology acknowledges previous research which emphasizes the importance of understanding both the technical capabilities and limitations of eye tracking technology, in clinical and educational settings (8,17).

Both evaluated system setups facilitate real-time data collection and processing, highlighting its potential in dental diagnostics and education by enhancing the understanding of visual attention patterns (18). This technology promises to advance diagnostic precision and educational outcomes in dentistry, underscoring the importance of accessible eye tracking technologies.

The display monitor was chosen based on the clinical requirements for viewing OPG images, while the 60cm distance of the patient in front of the screen is especially significant in daily clinical practice, where space constraints and the need for natural interaction with diagnostic images necessitate a setup that is both practical and effective. This flexibility is crucial in a clinical setting, where the natural variability in the positioning and posture of practitioners as they engage with radiographic images can otherwise impact the quality of data collected.

The second system under investigation was Webgazer.js, an open-source eye-tracking library that leverages webcam data to provide real-time gaze prediction on a web page. It uses common web technologies such as JavaScript and HTML, making it easy to integrate into web applications. The browser-based eye-tracking library Webgazer.js is designed to function without the need for specific eye-tracking hardware, utilizing the laptop’s built-in or externally mounted camera to monitor gaze patterns. Therefore, a MacBook Air and an IPEVO V4K external camera with Firefox browser were used to establish a reliable eye-tracking setup. The MacBook’s bright display and widescreen aspect ratio enhance visibility in varied lighting conditions found in dental clinics. The high-resolution IPEVO camera captures detailed images, vital for precise eye tracking in a cost-effective manner. Due to its support for web technologies and performance as well as ease of selecting alternate camera input, Mozilla Firefox was used as the preferred browser for the Webgazer.js application.

The illuminance at the study site is maintained between 100-200 lux to reduce monitor glare and reflections, thus minimizing eye strain for participants and ensuring the clarity of the OPG images based on recommendations evaluated in previous research(19). This level of illumination is essential for consistent visual perception during all sessions.

On the Peye Tracker system, participants with contact lenses (ac) showed a notably higher calibration accuracy compared to both participants wearing glasses (ag) and those without any visual aids (na) (Image 4a). This outcome suggests that the Peye Tracker may be better attuned to detecting and tracking the gaze of users with contact lenses, potentially due to the minimal interference they present to the tracking technology.

No significant statistical difference was observed between participants without visual aids and those wearing glasses, indicating that glasses may not adversely affect the calibration accuracy of the Peye Tracker. However, comparing these results with Image 4b suggests that the Peye Tracker could face challenges in precise eye detection, possibly because of glare from metallic frames on glasses.

Conversely, the Webgazer.js system presented a different pattern of accuracy across the groups. The group wearing glasses (ag) achieved significantly higher calibration accuracy compared to the group with contact lenses (ac). This variance could imply that the Webgazer.js system is more adept at accommodating the reflections or refractions introduced by glasses, or it may suggest that the system’s software algorithms are better optimized for users wearing glasses.

The analysis of heatmap accuracy in this study highlights significant challenges, primarily the low resolution of detail in identifying clinically relevant areas. This limitation underscores a potential shortfall in the capability of economical eye-tracking devices to deliver the level of precision necessary for dental diagnostics (20).

The granularity required to pinpoint areas of interest within dental imagery is crucial, and the current performance suggests that these devices may not be adequate for professional applications (7,17).

This limitation suggests that the precision of low-budget eye-tracking devices may not meet the stringent requirements necessary for clinical diagnostic tasks. The inability to accurately identify areas of interest could significantly impact the utility of these devices in a clinical setting, where the fine-grained analysis of radiographic images is vital (21).

The importance of the clinical setup for conducting eye-tracking research in dentistry cannot be overstated. Factors such as the seating arrangement of participants, the room’s illuminance, the proximity to the eye-tracking apparatus, and the use of visual aids like prescription glasses or contacts are pivotal factors that can impact the integrity of the data collected (8,17) These environmental and ergonomic considerations are paramount in creating conditions conducive to accurate tracking of visual attention and, consequently, the generation of precise heatmaps.

These preliminary findings raise the question of whether the observed inaccuracies are a result of the experimental setup or inherent limitations of the eye tracking technology employed. It should be investigated whether adjusting the clinical setup by optimizing seating positions, lighting conditions, or eye tracker placement could enhance the accuracy of these devices.

Moreover, the deficits observed might indicate a fundamental performance ceiling of low-cost eye trackers.

To address these uncertainties, further research is necessary. Subsequent studies should aim to systematically investigate the impact of various clinical setup parameters on the accuracy of eye tracking devices (18). Only through rigorous exploration one can ascertain whether modifications to the experimental environment can mitigate the current limitations or whether investment in more advanced eye tracking technology is necessary for clinical applications in dentistry.

## Conclusions

It was observed that the accuracy of the Peye Tracker remained uncompromised with the use of contact lenses, and the Webgazer.js system was highly effective for participants wearing glasses. While the Peye Tracker and Webgazer.js systems show promising potential to improve diagnostic accuracy and educational methods in dentistry, challenges remain in their integration into clinical settings, which include complex technical requirements, intricate setup procedures, and accuracy limitations that detract from their utility in real-world dental practices.

## Figures and Tables

**Table 1:** Setup of the low-budget, commercially available eye-tracking systems (Peye Tracker and Webgazer.js).

Feature	Peye Tracker	Webgazer.js
Compatibility	Works with Raspberry Pi; can be mounted on display screens or flat surfaces	Utilizes laptop's built-in or external camera; no need for specialized hardware
Hardware	Raspberry Pi Version 4B in combination with viewing monitor and Windows PC	2023 MacBook Air with 13-inch Retina display combined with an external camera IPEVO V4K
Software	Peye Tracker Client software version 5.3	Browser-based, operates through Mozilla Firefox Version 120.0
Operating System	Peye Tracker OS for Raspberry Pi Windows 10 Professional Edition	Mac OS Sonoma 14.2
Display	24-inch Asus VE 248 screen, 1920x1080 resolution, 60 Hz refresh rate, RGB color format, up to 250 cd/m² brightness	2560x1600 pixels resolution, 60 Hz refresh rate, RGB color format, 400 cd/m² luminance
Frame Rate	Capable of tracking at 50 fps	external camera supports 4k resolution at 30 fps
Setup	Guided by manufacturer specifications, LAN/WLAN connection between systems required	Follows developer guidelines, including setup of SQL database
Calibration	9-Point Calibration	9-Point Calibration followed by central point fixation
Data Capture	Wirelessly records gaze data to CSV file using Peye Tracker Client	Recording of gaze data to SQL database (separate setup)
Clinical Environment Adaptation	Allowing head movement within an 18.5 cm by 30 cm area without losing accuracy	Setup to mimic clinical practice with considerations for environmental illuminance and participant comfort

**Table 2:** The study categorizes participants by age, gender, correction aids and visual acuity (data provided by ophthalmologists) into three groups: no aids, glasses, and contact lens wearers.

Age	Gender	Correction aids: na (no visual aids) ag (aids- glasses) ac (aids - contact lenses)	Visual acuity
24	m	ac	R: -1,25 L: -0,75
23	m	ac	R: -1,25 L: -1,25
24	m	ac	R: -4,75 L: -5,00
23	m	ac	not available
26	m	ac	R: -0,75 L: -0,75
27	m	ac	not available
33	m	ac	R: -9,00 L: -8,75
26	w	ac	R: -0,75 L: -0,50
32	w	ac	R: -1,00 L: -0,75
46	w	ac	R: +1,00 L: +1,00
24	m	ag	R: -4,75 L: -5,00
28	m	ag	R: -4,50 L: -4,00
34	m	ag	R: -0,50 L: -0,75
27	m	ag	R: -1,25 L: -1,25
26	m	ag	not available
23	m	ag	R: -1,25 L: -1,25
30	m	ag	R: -4,50 L: -4,00
32	w	ag	R: -1,00 L: -0,75
26	w	ag	not available
48	w	ag	not available
30	m	na	not available
50	m	na	not available
24	m	na	not available
23	m	na	not available
34	m	na	not available
23	m	na	not available
29	m	na	not available
26	w	na	not available
31	w	na	not available
26	w	na	not available

## Data Availability

The datasets used and/or analyzed during the current study are available from the corresponding author.
